# Mineral Resource Dilemma: How to Balance the Interests of Government, Local Communities and Abiotic Nature

**DOI:** 10.3390/ijerph110908632

**Published:** 2014-08-25

**Authors:** Nataliya Nikitina

**Affiliations:** License Department, LLC Intergeo Managing Company, Moscow 125009, Russia; E-Mail: Nikitina-NK@yandex.ru; Tel.: + 7-495-287-29-51; Fax: +7-495-287-29-51

**Keywords:** mineral resource dilemma, governments, subsoil users, local communities, abiotic nature, environment, social conflicts

## Abstract

It is noted that over the last few years the implementation of several mineral exploration, development and mining projects has been suspended and even completely stopped due to resistance from local communities. The key concerns of local residents typically include perceived or real impact of mining enterprises on the environment, unfair distribution of profits from mining and exploration activities, insufficient contributions to local government budgets and lack of transparency regarding ultimate ownership of companies conducting exploration and mining. The article looks at social conflicts of this kind and suggests some alternative solutions that could prevent such conflicts at the stage of granting exploration and mining rights.

## 1. Introduction: Social Conflicts in Subsoil Use

Subsoil use is activity pertaining to geological exploration, mining, including utilization of waste products of mining and related processing industries, construction and operation of underground facilities unrelated to mineral production, special protection of geological features of scientific, cultural, aesthetic, sanitary and other value (scientific and training grounds, geological reserves, caves and other undergrounds areas), sampling mineral, paleontological, and other geological materials for collection purposes.

Over the past ten years, a number of exploration and mining projects in various countries have been delayed or stopped as a result of strong opposition from local communities. Early in July 2013 the Argentine Government decided to cancel its agreement with the Canadian gold mining company Osisko (one of Canada’s largest gold mining companies) to develop a gold mining project in the north-west of the country after the protests of environmentalists, despite significant social and economic consequences. Local authorities of the La Rioja Province were not able to achieve an agreement with local population community that resisted the project in the last two years with support from Greenpeace. The main ecologic concern was the use of cyanide and a large volumes of water for the precious metal mining.

In April 2013, due to the local community protests, the Gaychursky ore-extraction and processing company located in the Zaporozhye region (Ukraine) lost its license to mine iron ore on the Gulyaypolsky deposit that was granted at the end of 2012. Also in Ukraine, since January 2013, when Shell and the local company “Nadra Yuzivska” signed a joint venture agreement to develop the Yuzivska shale gas field, the local community of the Donetsk and Kharkov regions has been protesting against Shell’s plans to extract gas in the region. The main concern is about the risk of negative environmental impacts on those areas in case of hydraulic fracturing. In addition, according to experts from the National Ecological Centre of Ukraine shale gas extraction poses a significant danger to the “Svyatye Gory” national park. The proposed project will also be a breach of Bern Convention on the Conservation of European Wildlife and Natural Habitats, Bonn Convention on Migratory Species, and the African-Eurasian Waterbird Agreement. Facing growing protests company officials indicated that the company is prepared to abandon the project if the public continues to be against the development of the field.

On 19 August 2013, British police dispersed hundreds of protesters who were blocking access to an oil exploration site in rural England in an intensification of month-long standoff over the shale gas extraction industry in Britain. A total of 36 people were detained, both in the village of Balcome and in London, during the first of two days of “direct action” against hydraulic fracturing, which protesters fear may trigger small earthquakes and pollute water supplies. Hundreds of protesters converged on the West Sussex village and scuffled repeatedly with around 400 police.

In order to stimulate a U.S.-style production boom and offset decreasing North Sea oil and gas reserves, the UK government has backed hydraulic fracturing as an “energy revolution” that can create jobs and lower energy prices. British gas imports have so far mostly come from Norway and, increasingly Qatar. Last year it imported around 50 billion cubic meters of gas via pipelines and liquefied natural gas (LNG) ships. The country has potentially vast shale gas resources in underground rock formations; the government said last month there may be 1300 trillion cubic feet of gas present in the north of England alone.

Activists argue the government should invest in renewable energy rather than “fracking”, as the retrieval of gas and oil from rock by injection of high-pressure water, sand and chemicals is known.

In the near future, the company Kurilgeo, which is 100% owned by the Cyprus-based Solway Group, plans to start mining the Ainskoe gold ore deposit, located on Urup Island in the Greater Kuril Ridge, Russia, using heap leaching. Urup Island is considered to be the most important habitats of rare marine animals including sea otters, Kuril seals, and sea lions, all of which have also been listed on the Red List of endangered species. The main breeding grounds of the animals are located in the immediate vicinity to the Ainskoe deposit. Urup Island has been named a special area of key importance for the conservation of the Kuril sea otter population by the Red List of the Sakhalin region and the Red List of the Russian Federation. The Red List of the Sakhalin region has also recommended the creation of a special protected natural reservation on Urop Island. The island had been a natural reserve from 1958 until 2003, when this status was revoked. Local residents have appealed to the Prime-Minister of Russian Government Dmitry Medvedev and the Minister of Natural Resources and Environment, Sergey Donskoy, with a request to stop the proposed gold mining project and to create a specially protected natural reservation on the island.

Also, in Khakassia (Russian Federation), local residents have been actively protesting against the planned construction of the Arshanovsky coal mine on the Beysky field. The planned annual capacity of the mine is 5 million metric tons of coal during the first phase and 10 million metric tons in the second phase. The Arshanovsky coal mine will be located 1 km from local towns and, according to residents, it will become impossible to live in its proximity due to the foreseen high concentrations of gas and dust, on the site, which a planned depth of 200 m. Under these conditions adjacent agricultural lands will be almost impossible to cultivate, which in turn might lead to the disappearance of four ancient villages populated by natives at the moment. The mining project might also significantly pollute the Abakan River, which is the source of drinking water for local cities and towns with a total population of some 300,000 people. Local residents have held a number of meetings in local villages and towns and are preparing a joint petition to the President of the Russian Federation with demands to stop development of the mine.

Often local populations protest not only against currently running exploration and mining projects, but also against proposed auctions and tenders that distribute the rights to eventually explore and develop various deposits of mineral resources (Table 1). In early July 2013, the Transbaikalian Mineral Resource Management Agency has announced the auction for the right to explore and mine alluvial gold in the basin of the Kirkun River of Kyrinsky District in the Russian Federation. A site area was 7.7 km^2^ with total expected gold resources of 23 kg, and the minimum (start-up) rate of subsoil use was a one-off payment to the government of 77 thousand rubles (around U.S. 2500). Representatives of environmental organizations—the International Coalition “Rivers Without Borders” and the Amur branch of the World Wildlife Fund (WWF)—appealed to the Minister of Natural Resources and Ecology of the Russian Federation to cancel the auction due to the high natural value of Amur river basin and include this and other areas situated in the transboundary basin of the river Onon in the Federal Fund of Reserve Subsoil Areas.

In Russia a highlight of similar protests has been confrontation of local community and local authorities in the Voronezh region in regards to the proposed development of copper-nickel deposits in the area. In 2011 the federal government decided to organize a tender to grant rights to explore and mine Elkinsky and Elansky copper and nickel deposits. Subsequently a number of protests were organized by local residents of Voronezh and surrounding areas against the exploration and development of these fields. Several social movements have been formed—such as “In Defense of Khoper” and “Green Ribbon”—including the unnamed action groups collecting signatures against the nickel project.

**Table 1:** Announced mineral resources auctions that mineral drew protests from the local communities in Russia.

Deposit/Field and Location	Action Announcement Date	Type of Mining Rights Offered	Area, Square Kilometers	Inferred Mineral Resources	One-Off Subsoil Use Payment, US$	Possible Dangers to the Environment and to Health of Local Population Caused by Mining Operation (According to Local Population and Environmental Organizations) and Claims Advanced by the Local Communities
Tsagan plot, Transbaical Territory	10 July 2013	Exploration and production of alluvial gold	7.7	23 kg	2567	Destruction of natural landscapes. Pollution of the Onon river basin Absence of socio-economic benefits for the local population in light of insignificant reserves of alluvial gold It is proposed to cancel the auction and to include the Tsagan plot and other subsoil plots of alluvial gold in the transboundary Onon river basin in the Federal Fund of Reserve Subsoil Areas
Shapsugsky plot, Krasnodar Territory	25 June 2013	Geological survey, exploration and production of natural cement rocks	24.84	Р_3_—160 M MT	20,000	The destruction of forests and natural landscapes of the Skabido and Abin rivers with their feeders that are favorite recreation areas of locals and tourists near the Shapsugsky village. The area also includes historical and cultural monuments, namely three ancient dolmens in a good state of preservation, a mud volcano, and remains of ancient fortress on the Ostray mountain, as well as the famous recreational site Romashkina Polyana. It is suggested to cancel auctions and create a specially protected natural territory near the Supsugsky village
Erivansky plot, Krasnodar Territory	25 June 2013	Geological survey, exploration and production of natural cement rocks	8.38	Р_1_—60 M MT	10,000	
Abinsky plot, Krasnodar Territory	25 June 2013	Geological survey, exploration and production of natural cement rocks	2.55	Р_3_—40 M MT	6900	

The population is seriously concerned with its own health and the safety of the recreational resources, unique Voronezh black soils, the purity of surface and ground waters of the river Khoper, recognized by UNESCO as the cleanest river in Europe, the Khoper Reserve with plants and animals listed in the Red Book, including the state of nature as a public domain.

However, public administration bodies in the sphere of subsoil resources believe that the possibility of profitable nickel mining in the Norilsk mining district in northern Russia is almost exhausted, and the inferred reserves of the Voronezh region in the event of positive results after exploration activities can be implemented in reserves of nickel, copper and cobalt, the largest in Europe, and the future mine will provide opportunities for jobs and development of the social sphere.

In accordance with the contest results dated 22 May 2012 the winner was the Mednogorsk Copper and Sulfur Plant—a subsidiary of the Ural Mining and Metallurgical Company. On 26 July 2012 the winner was issued the licenses for subsoil use. Geologically, the license areas are located in the Elansky and Uvarovsky mining district of the Kalach-Ertilskaya zone of the Voronezh crystalline core-area. Inferred resources of categories P_2_ + P_3_ of the Elkinsky license area amount to 993,800 tons of nickel, 33,900 tons of cobalt, 129,600 tons of copper, Elansky—54,100 tons of nickel, 5.6 tons of copper, 1.7 tons of cobalt P_1_ and 1,753,500 tons of nickel, 209,300 tons of copper, 53,300 tons of cobalt in category P_2_ + P_3_.

In accordance with the terms of subsoil use, exploration of these areas should be completed in May of 2020, a technical development project should be drawn up by May of 2012, the construction of infrastructure facilities of the mining enterprise should start in 2022, and in 2027 the mining enterprise should be put into service and by 2028 it should reach the design capacity.

In June 2012, in an attempt to solve the ethical dilemma using the existing legal instruments, the social organizations appealed to the regional court to obtain the right to hold a referendum on the development of copper and nickel deposits of the Voronezh Region, but the court denied the claims, explaining that these areas belong to the subsoil plots of federal importance, and the right to dispose of them belongs to the federal authorities, and is not under the joint competence. Considering that such a decision of the regional court does not take into account the other provisions of the Russian Federation Constitution, the social activists appealed to the RF Supreme Court.

On 14 September 2012 the RF Supreme Court refused to hold a referendum on the issue of the nickel deposits development in the Voronezh Region. The Court agreed with the decision of the Voronezh Regional Court and noted that the Russian Federation has the exclusive right to use these deposits. On 22 July 2013 another protest took place in the Voronezh Region. More than a thousand protesters went to Elansky field, where the temporary settlement of geologists was situated. The protesters broke a fence surrounding the exploration site and construction trailers, and set on fire to two drilling rigs, each worth US $1,000,000.

## 2. The Ethical Analysis of Conflicts 

Similar examples in other countries with the participation of different peoples and local communities indicate some real trends of increasing negative attitude of the local population toward any exploration and mining work, regardless of exploration methods, mining systems and environmental protection measures. In particular, mining activities are viewed more critically in areas where people strongly rely on ecosystem services or have suffered from negative environmental impacts before. People are always reluctant to live close to areas with a significant level of mining activities. Perceived and actual environmental impacts created by mining operations are one of the most frequent causes for the local population to oppose new projects in their region. In many places communities report a lack of financial benefits to local business in spite of massive profits for mining companies and royalties for government.

However, it cannot be denied that the population growth, social progress and the unlimited desire of the population to increase its living standards and comfort require permanent economic advancement accompanied by increasing production and consumption non-renewable mineral resources production. This is also illustrated by the world production statistics (Table 2).

**Table 2:** Total world main mineral resources production (source: BP Statistical Review of World Energy, June 2013; World Coal Institute, 2012; World Nonferrous Metal Statistics 1986–2005; GFMS Gold Survey, 2012)

Years	Production					
	Natural gas (B CM)	Oil (M MT)	Coal (M MT Oil Equivalent)	Uranium (MT)	Gold (kg)	Nickel (T MT)
1970	1021	2358.0	-	-	-	-
1980	1456	3092.0	2805.0	-	-	-
1985	1676	2797.0	-	34,936	1,606,573	771.6
1990	2000	3175.0	2677.0	49,728	2,149,276	894.5
1995	2141	3286.0	-	33,084	2,175,279	1030.4
2000	2436	3611.8	-	35,221	2,565,884	1223.8
2001	2493	3601.6	-	36,363	2,543,873	1284.0
2002	2524	3584.2	2401.9	36,400	2,537,657	1303.1
2003	2620	3701.1	2572.7	35,812	2,538,438	1349.5
2004	2691	3877.0	2781.3	40,551	2,496,000	1355.0
2005	2780	3906.6	2942.4	41,827	2,550,000	1383.9
2006	2880	3916.2	3100.7	-	2,482,000	1397.0
2007	2943	3904.3	3211.1	-	2,476,000	1440.3
2008	3054	3933.7	3324.2	-	2,408,000	1484.9
2009	2969	3831.0	3354.3	-	2,589,000	1530.9
2010	3192	3913.7	3542.7	-	2,689,000	1590.4
2011	3291	4019.0	3759.1	-	2,694,000	1800.0
2012	3364	4119.0	3845.3	-	2,700,000	2100.0

Almost all the specialists in the sphere of natural resources law while analyzing the legal status of natural sites emphasize that the concepts “earth”, “mineral resources”, “water”, “forest” have a deep moral nature and cannot be anything else but the national property. Without going into discussion about the problems of title to subsoil it should be noted that in most of countries subsoil, including mineral resources contained therein, energy and other resources are state property [1].

Therefor the mineral resource base as a state property and public domain can be described as a non-renewable natural object, the right to use which may be granted to individuals and legal entities on paid and fixed-term conditions with the obligation of the user to comply with licensing terms and conditions. In this case process of involving certain resources into exploration and mining reserves should ensure public interests of both current and future generations.

Recognition of subsoil and mineral resources as state and public property in a constitution (*i.e.*, Germany, Greece, Spain, Italy, Norway, Russia, Sweden) puts an obligation on the government to ensure balanced and efficient use of mineral resources to the public benefit by designing and implementing specific policies and strategies for the use and replacement of mineral resources in a country. Governments need to find cost-effective solutions to ensuring a sustainable development of mineral resource base and preservation of the environment in conditions of uneven geographical distribution of mineral deposits both in Russia and worldwide. Sustainable mining is a theoretical, but highly unlikely, possibility. The use of non-renewable resources—such as metals and minerals—can be sustainable if the use is declining, and the rate of decline is greater than the rate of depletion.

In Russia subsoil, including the subsoil domain and mineral resources contained therein, energy and other resources are state property. Issues of ownership, use and disposal of subsoil shall fall under the joint jurisdiction of the Russian Federation and the subjects of the Russian Federation. Mineral and other subsoil resources produced under license terms may have the status of federal property, the property of the Russian Federation sub-divisions, municipal, private or any other property status.

The examples of the Elkinsky and Elansky copper and nickel deposits described above fall under the jurisdiction of the federal government according to the “On subsoil” law of the Russian Federation and therefore decision to grant a mining license for these deposits as well as responsibility for any consequences lies with the federal government as well. It is a pity in this case the federal government of Russia could not foresee such a negative reaction from local population and thereafter simply left the mining company to deal with the local community. It is also hard say if when making the decision to grant the mining license for these deposits the government had also taken into account article 9 of the Constitution of the Russian Federation (“Land and other natural resources shall be utilized and protected in the Russian Federation as the basis of life and activity of the people living in corresponding territories”) and article 36 (“Possession, utilization and disposal of land and other natural resources shall be exercised by the owners freely, if it is not detrimental to the environment and does not violate the rights and lawful interests of other people”) [2].

The problem of the social (non-) acceptance of the mining industry is relatively new, but has been quite widely discussed at least since the 90s. It is noted up to the present day its focus was on three core themes of Community and Environmental Sustainability, Operational Effectiveness, and Social Responsibility of Business [3,4,5,6,7,8]. Relations in complicated system “Abiotic nature—the state as the owner of the subsoil—the mining company” were not considered.

In the reality there is another silent participant of the conflict—abiotic nature. It is important to recognize and respect its rights (or “quasi-rights” as a correlate of legal rights), and support its “interests” in practical discourses and institutes created by man.

Any mining activities have a negative impact on natural landscapes, disturb groundwater hydraulics, contaminate soil, subsoil and underground water, reduce geodiversity (geodiversity is the natural range (diversity) of geological (bedrock), geomorphological (landform) and soil features, assemblages, systems and process. Geodiversity includes evidence for the history of the Earth (evidence of past life, ecosystems and environments) and a range of process (biological, hydrological and atmospheric) currently acting on rocks, landforms and soils [9] and *etc.* The consequences of exploitation and destruction of abiotic nature is not perceived as a real threat to our existence, such as nuclear war. Firstly, the consequences are perceived to be far away in the future and secondly, mining creates illusion of a value-add activity for the population. Therefore, the conservation of abiotic nature is by no means evident and important in the eyes of many people. As Grey notes the expression “Save the Dolphin” is always likely to have greater appeal to the public than “Save the Drumlin” [10].

Nevertheless, the demands to stop or prevent mining activities are obviously inadequate. There is a need to find an adequate solution that would be based on ethical principles and also take into account commercial rationale. Most countries, especially those that possess significant mineral resource bases, face a geoethical dilemma, so-called the mineral resource dilemma. In most of examples described above the state could not foresee social conflicts, and as a result the desperate local population or mining companies have to search for solutions separately. Since geoethical dilemmas only arise if one party in a conflict will incur a loss in any case, a solution to the issue will be based on the ethical grounds and involving a “lesser evil” principle.

## 3. The Mineral Resource Dilemma and Feasible Solutions 

Typically the mineral resource dilemma looks as follows:

Sooner or later companies that hold exploration or mining rights need to obtain consent (formal or informal) of the local population for exploration and mining activities in the area. Both parties have to make a decision:

- The local community does not argue against the government decision to grant the right to explore/mine/extract mineral resources in a certain area and in 8–10 years the local budget will receive additional income, the size of which will depend on revenues and costs of the mining business, especially environmental remediation expenditures, community-related expenditures. In this case the environment and subsoil will suffer a certain degree of degradation;
- The local community lobbies against exploration and production of mineral resources and a result the mining license is revoked by the government or forfeited be the mining company. In this case the local budget will obtain no additional revenue and no environmental damages will be suffered. Alternatively instead of forfeiting the license, the mining company can decide to substantially increase its environmental remediation expenditure (to the satisfaction of the local community) in which case the environmental and community related damages are minimized as well as the additional revenues of the local budget (Table 3).

A solution to the dilemma is determined to a certain extent by the goals and interaction strategies of parties involved. If each party is only considered its own goals (profits maximization of a company or nature preservation at any cost), alternative # 1 will be the best for the local community and alternative # 2 for the mining company. But from a joint point of view, if the mining company and the local community are aware of limited uneven geographical distribution of mineral resources, growing consumption of mineral resources by society and need for economic development, while preserving (to the extent possible) the environment from the negative effects of mining, it would be the best to act together using alternative # 3 and # 4. In this case, the solution to the dilemma will be found depending on, firstly, demands of the local community, secondly, on the amount of environmental and social oriented expenditures that the mining business is prepared to bear.

**Table 3:** Matrix of possible solutions to the mineral resource dilemma and their consequences.

Feasible Solutions	Consequences			
	Local Community against the Proposed Mining Activity		The Local Community is Indifferent to the Proposed Mining Activity	
Mining business does not take into account protests or social needs of the local community	Alternative # 1	Mining company has to cancel the project completely and suffers a direct loss	Alternative # 2	Mining company obtains a maximum mining profit
		Environment and abiotic nature completely preserved		Environmental and subsoil degradation
		Local budget does not receive any mining revenues		Maximum mining revenues to the local budget
Mining business incurs substantial additional environmental remediation costs and community oriented expenses	Alternative # 3	Minimal profit for mining business due to maximized environmental expenditures	Alternative # 4	Moderate profit for mining business due to obligatory environmental remediation and community related expenses
		Minimal damage to the environment and abiotic nature		Limited damage to the environment and abiotic nature
		Moderate local budget revenues		Moderate local budget revenues

At the same time any dilemma participants cannot be sure that the other side will meet its obligations during the agreed mining period. If the mining rights are revoked by authorities for certain reasons or in case of mining company deciding to forfeit the rights, any legal or moral obligations of the mining company regarding the environment or local community will fall away. Also there is no guarantee that the requirements of the local population will not change in the future.

The matrix above (Table 3) shows the final solution to the mineral resource dilemma. However, the origin of the mineral resources dilemmas and its consequences (protests of the local communities, economic losses of business, damage to the environment, and others) are determined, primarily, by the decision of the state to conduct a geological survey, and organize exploration and production of the subsoil plots. From the geoethical point of view such decisions should be made based on the geoethical imperative of sustainable development being determined within the tripartite system of abiotic nature, man and society [11]. In which case it is important to ensure:

- the human right to a healthy and productive life in harmony with nature;
- equal opportunities for the development and preservation of abiotic nature for present and future generations, including mineral resources, useful properties of subsoil, landscapes *etc.*;
- socio-economic development aimed at improving the quality of human existence within limits of the economic capacity of geological systems and sites;
- elimination of causes of negative mining impact and not is consequences;
- development of geoethical consciousness and mind set as well as geoethical education system.

In practice, this means that the process of distribution mining rights should be preceded by establishing programmes of sustainable development and replenishment of mineral resources that are based on current and forecasted consumption and production levels and available information about possible decrease/increase of mineral resource base. These programmes should also take into account the needs and objectives of the government as well as of local communities. Before any mining rights are given to any company it is important to address of the concerns of local population that are listed below:

- Is the development of the particular mineral deposit really necessary for the economy?
- What short-term and long-term benefits will be received by the federal and local governments on case of mine development?
- What possible dangers and threats to public health and living conditions can arise as a result of the mining activity?
- What objects, components, elements, systems of the environment will be lost forever or will undergo degradation in case of mine development?
- What is the balance between the economic benefits from mining to the state and population and caused negative environmental impact?
- What specific activities are planned for the remediation, rehabilitation and restoration of land areas and other natural features damaged in the course of the mining activity on the site? What is the timeline and certainty of these activities being implemented?

Another important issue to be considered is the fair distribution of the benefits from mining. The federal law “On the Federal budget for 2013 and the planning period of 2014–2015” provides for the following guidance on the income distribution from mineral resources exploration and production (Table 4).

**Table 4:** Guidance on income distribution from the mineral resources exploration and production between the various budgets of the Russian Federation for 2013 and the planning period of 2014 and 2015 (%).

Item of Income	Federal Budget	Budgets of Constituent Entities of the Russian Federation
One-off subsoil use payments in the case of onset of certain events stipulated by a license (except for subsoil plots containing deposits of diamonds, and local-significance deposits of mineral resources)	100	-
One-off subsoil use payments in the case of onset of certain events stipulated by a license for use of local-significance subsoil plots	-	100
Mineral resource recovery tax (combustible natural gas)	100	-
Mineral resource recovery tax for hydrocarbon raw materials (except for combustible natural gas)	100	-
Mineral resource recovery tax (except hydrocarbon raw materials, natural diamonds and commonly occurring mineral resources)	40	60
Mineral resource recovery tax for commonly occurring mineral resources	-	100
Mineral resource recovery tax for natural diamonds	-	100
Regular subsoil use payments	40	60

As shown in Table 4, most of the income from the distribution of the mining rights at the initial stage flows into the federal budget, including one-off subsoil use payments in the case of onset of certain events stipulated by a license (except for subsoil plots containing deposits of diamonds, and local-significance deposits of mineral resources). Budgets of constituent entities of the Russian Federation would only be entitled to regular subsoil use payments during geological survey and exploration (60% of the regular payments that are very low and to a share of mineral resources recovery tax, in production phase 60% of the mineral resource recovery tax for solid minerals and 100% in case of commonly occurring mineral resources). The issue is that the initial one-off subsoil use payments are made to the federal budget at the time of the auction process while the payments to the local budget only start flowing in during the production phase which is usually 7–10 years later that the initial auction. In the meantime local population can already be faced with negative consequences from the mining operation including destruction of the environment overall and the abiotic nature in particular in the course of the exploration, construction of infrastructure and mining. Such imbalance in the distribution of the revenues between the federal budget, budgets of constituent entities of the Russian Federation and local budgets causes significant concerns to the local population and can further impact the relationship between the local community, mining companies and local government.

This imbalance leaves the local population asking the following questions: who is the ultimate shareholder(s) of the mining company and where has the company been domiciled? First of all, the answers are strictly confidential. Secondly, in the event when the mining company is registered overseas, the governing body will have to consider whether the deposit in question has a status of “federal importance” which in turn puts certain restrictions on non-Russia domiciled companies being able to operate it. Thirdly, it should be noted that most of the mining companies operate in Russia at the moment are domiciled overseas with the exception of “ALROSA”. Overseas domiciliation is primarily used to fond and obtain cheap loans, without which it is impossible to develop mining projects in Russia, and in order to avoid a hostile takeover. However, regardless of domiciliation specific subsoil use payments and the mineral resource recovery tax flow to the budgets of the Russian Federation in any event. In this situation, the government needs to foster a business environment in which companies can maintain and develop their business and also provide support and care to the abiotic nature and local population.

On the other hand a mining company that intends to apply for a mining license for a particular deposit and plans to operate in a certain area, needs to have a common strategy of conflict prevention, including situation analysis, stakeholder engagement and integrated impact assessment. To ensure economic feasibility, profitability and continuity with respect to mining activities, the business needs to engage and secure “approval” of local community. 

There are now many successful big and small corporations (CJSC Petropavlovsk Managing Company, Kinross Gold Corporation, *etc.*) working hand in hand with local communities, environmentalists, civil society groups and governments. Their purpose is not only the profit from mining, but also to improve quality of life of local population, to support local townships, to provide alternative job placement after closing of mining projects and to restore land areas and other natural features damaged by mining activities to a condition suitable for further use, while conserving the environment and preserving cultural heritage.

## 4. Conclusions

It is essential recognize that the need for a healthy environment is a basic need what if using Maslow’s hierarchy comes after safety needs (protection from elements, security, order, law, limits, stability, freedom from fear) and is followed by social needs (belongingness, affection and love) of a human being:

1. In order to avoid conflicts with the public and local populations during mining activities, the government should only allocate the mining rights after thorough analysis of forecasted levels of production and consumption of the mineral resource, detailed review of possible economic and social development of a particular region, understanding specific goals and objectives of the government and the local population that will be achieved a result of a mining project and analysis of possible social and environmental risks.
2. Distribution of mineral resources revenues between budgets should be transparent and equitable, so that local population is appropriately compensated for environmental degradation, decline of bio- and geodiversity, and deterioration of population health.
